# Conditions for the Successful Integration of an eHealth Tool "StopBlues" Into Community-Based Interventions in France: Results From a Multiple Correspondence Analysis

**DOI:** 10.2196/30218

**Published:** 2022-04-22

**Authors:** Kathleen Turmaine, Agnès Dumas, Karine Chevreul, PRINTEMPS Consortium

**Affiliations:** 1 Université Paris Cité, ECEVE, UMR 1123, Inserm Paris France; 2 Assistance Publique-Hôpitaux de Paris, URC Eco Ile-de-France, Paris, France Paris France; 3 Assistance Publique-Hôpitaux de Paris, Hôpital Robert Debré, Unité d'épidémiologie clinique Paris France; 4 See Authors' Contributions

**Keywords:** eHealth, internet-based intervention, community participation, health promotion, prevention, mental health

## Abstract

**Background:**

For over a decade, digital health has held promise for enabling broader access to health information, education, and services for the general population at a lower cost. However, recent studies have shown mixed results leading to a certain disappointment regarding the benefits of eHealth technologies. In this context, community-based health promotion represents an interesting and efficient conceptual framework that could help increase the adoption of digital health solutions and facilitate their evaluation.

**Objective:**

To understand how the local implementation of the promotion of an eHealth tool, StopBlues (SB), aimed at preventing psychological distress and suicide, varied according to local contexts and if the implementation was related to the use of the tool.

**Methods:**

The study was nested within a cluster-randomized controlled trial that was conducted to evaluate the effectiveness of the promotion, with before and after observation (NCT03565562). Data from questionnaires, observations, and institutional sources were collected in 27 localities where SB was implemented. A multiple correspondence analysis was performed to assess the relations between context, type of implementation and promotion, and use of the tool.

**Results:**

Three distinct promotion patterns emerged according to the profiles of the localities that were associated with specific SB utilization rates. From highest to lowest utilization rates, they are listed as follows: the privileged urban localities, investing in health that implemented a high-intensity and digital promotion, demonstrating a greater capacity to take ownership of the project; the urban, but less privileged localities that, in spite of having relatively little experience in health policy implementation, managed to implement a traditional and high-intensity promotion; and the rural localities, with little experience in addressing health issues, that implemented low-intensity promotion but could not overcome the challenges associated with their local context.

**Conclusions:**

These findings indicate the substantial influence of local context on the reception of digital tools. The urban and socioeconomic status profiles of the localities, along with their investment and pre-existing experience in health, appear to be critical for shaping the promotion and implementation of eHealth tools in terms of intensity and use of digital communication. The more digital channels used, the higher the utilization rates, ultimately leading to the overall success of the intervention.

**International Registered Report Identifier (IRRID):**

RR2-10.1186/s13063-020-04464-2

## Introduction

### Digital Health and Community-Based Health Interventions

For over a decade, digital health has held promise for enabling broader access to health information, education, and services for the general population, all at lower cost [1,2]. The unbridled development of the digital health market has led to more than 320,000 health apps currently available, including around 10,000 specifically aimed at promoting mental and behavioral health. In spite of this, adoption rates have been relatively poor, limiting visibility to their use and overall impact on health [3,4]. In fact, it is difficult to sort through all the existing eHealth tools to find evidence-based solutions for user needs. Indeed, an increasing number of studies on the subject have shown mixed results, and currently, there is a certain disillusionment regarding its benefits [5-8].

In recent years, agencies and governments, such as the World Health Organization (WHO) or the US Federal and Drug Administration, have elaborated guidelines and regulations in order to address the rather anarchic growth of eHealth technologies (eHT) [9-11]. However, it remains essential to conduct proper evaluations in order to assess overall eHT impact and benefits.

In this context, community-based health promotion (CBHP) represents an interesting and efficient conceptual framework, which could help increase the adoption of digital health solutions and promote greater opportunities for their evaluation [12,13]. While CBHP had been poorly theorized until very recently, it can be considered to have two defining characteristics. First, it is a community-based approach involving both professionals and local actors that emphasizes the holistic, preventative, and population levels rather than the pathogenic, curative, and individual levels. Importantly, it recognizes the social and organizational contexts in which people live, work, and interact [13,14]. Second, it relies “heavily on locally available channels of mass communication…with the potential to reach and change lifestyle behaviors of entire populations” [15]. Thus, through large-scale promotions, CBHP could allow eHT to reach more users, facilitating broader adoption and use within the population and thereby enhancing the level of evaluation results.

Since the successful development of the WHO Healthy Cities project [16,17], cities have been considered as one of the most suitable levels upon which CBHP can be established [18]. Therefore, environments in which CBHP is carried out should also be taken into full consideration. Further, it has been well-recognized that health status is influenced by social, economic, and environmental factors [19]. Thus, at the collective level, the availability of financial resources and health services, along with previous experience and expertise in implementing community-based interventions, can have a major impact on how CBHP is delivered [20-22]. Meanwhile, at the individual level, socioeconomic status (SES) and educational levels play a key role in how CBHP is accepted by the population. In digital health, particularly disparities in access and utilization patterns, notably across geographic and socioeconomic groups, have been highlighted [23]. Several studies have raised the risk of increasing health inequalities through the so-called “digital divide” [24-28].

### The Case of StopBlues: a French Self-Help Tool to Prevent Mental Distress and Suicide

StopBlues (SB) is a first-of-its-kind website and mobile application in France that aims at preventing mental distress and suicide [29]. It was originally created in 2018 within a CBHP intervention, carried out in a sample of municipalities and associations of municipalities (referred to in this paper as “localities”) where the tool was specifically promoted [29]. The idea was to anchor SB in local settings by offering the localities a ready-to-use eHealth tool, along with a promotional toolkit that they could customize and adapt to local needs. Similarly, they were invited to take part in the elaboration of the mental health resource locator included in the SB tool.

In each locality, an appointed delegate acted as the main point of contact with the research team and centralized the implementation of promotional actions locally. Two pre-experimentation meetings gathered the delegates to present the intervention and the SB tool and provide them with some suggestions regarding the implementation of promotion. Subsequently, the choice of promotional tools, the identification of public places and digital spaces for reaching out to the general population, as well as the launch date and the length of the promotion, were left to the discretion of the localities (Multimedia Appendix 2). This adaptable design added flexibility to the rigidity of standardized scientific experimentations [30,31]. Importantly, SB was included in a full evaluation program, in contrast to the limited research conducted via controlled trials or in real-world settings to evaluate the public health impact of eHT and mobile health applications, in particular [32,33]. The evaluation included a cluster-randomized, controlled trial (CRCT) [29] associated with qualitative research in order to better understand the implementation and identify the optimal conditions to ensure its utilization.

### Objective

The objective of this paper was to analyze and understand how the implementation of the promotion varied according to the characteristics of different localities and how those differences influenced the utilization rate of SB. To this end, we assessed whether there were distinct profiles of localities based on their characteristics and promotion implementation patterns and whether those profiles were associated with a specific usage intensity of SB.

## Methods

### Setting

**Figure 1:**
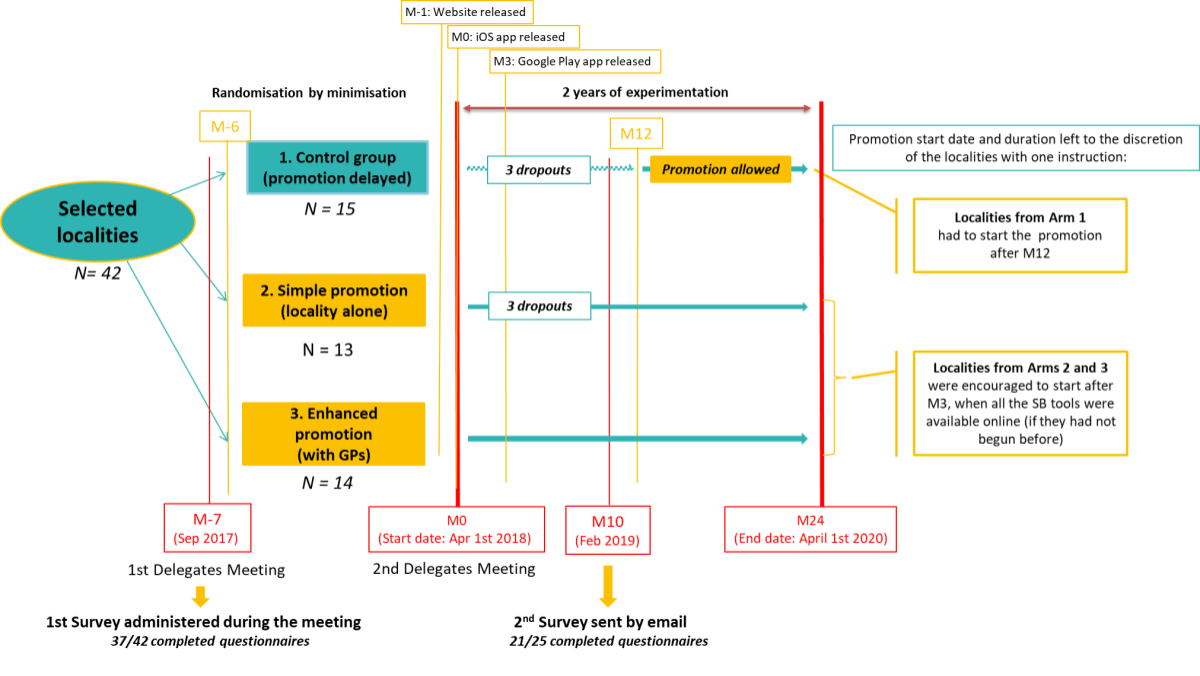
The intervention timelines. GP: general practitioner; SB: StopBlues.

A three-arm, parallel-group CRCT was conducted to evaluate the effectiveness of the promotion with before and after observation [29]. 42 localities volunteered to be randomly allocated to one of the following three arms with a ratio of 1:1:1 (Figure 1; Multimedia Appendix 2):

1. Arm 1: “the control group” with no promotion (n=15). The localities included in this group started the promotion of SB with a one-year lag.
2. Arm 2: “the simple promotion group” with promotion by the locality only (n=13).
3. Arm 3: “the enhanced promotion group” with promotion by the locality and through general practitioner (GP) waiting rooms (n=14).

In total, 27 localities from Arms 2 and 3 that implemented the promotion of SB directly were included in the present analysis.

### Multiple Correspondence Analysis

In order to identify the localities’ profiles and find a potential association with the utilization rates of SB, a multiple correspondence analysis (MCA) was run using contextual and implementation characteristics of the 27 localities included in the CRCT. An MCA is a descriptive and exploratory statistical technique that is often used to help with the organization and classification of large amounts of data [34-36]. Notably, it allows researchers to deal with the complexity of qualitative data without impoverishing the richness of the reality [37-39].

In the current study, the MCA technique was also selected for the purpose of detecting all the possible patterns of relationship among the considered variables through a geometric approach. Indeed, each variable (unit of analysis) is located as a point in a low-dimensional Euclidian space [36,37,40].

### Characteristics of the Localities Considered

All the characteristics included in the analysis were based on a review of the theoretical and empirical literature. Data collection included socioeconomic and demographic data from institutional sources, observations and discussions from the delegates' meetings, questionnaires, and web data extractions from SB analytic tools via Google Analytics (GA). The characteristics were divided into three sections:

1. The characteristics that were based on the pre-existing context.
2. The characteristics that derived from the program itself, its promotion, and implementation.
3. The utilization rate corresponds to the outcome variable of the promotion.

For the purpose of the MCA, these characteristics, detailed below, were transformed into a set of categorical variables. The characteristics from sections (1) and (2) were used as active variables that helped generate the MCA. The utilization rate was not used in the construction of the MCA but was added as a supplementary variable. The modalities of each variable are presented in Tables 1-3.

**Table 1:** Characteristics of the pre-existing context (context-based characteristics).

Variables and modalities (names in multiple correspondence analysis [MCA] if different)			Description	Frequency, n (%)
**Level of urbanization and socioeconomic context of the area**				
	Rural area & low socioeconomic status (SES)		Rural localities with lower levels of SES	6 (27.3)
	Urban area & low SES		Urban localities with lower levels of SES	8 (36.4)
	Urban area & high SES		Urban localities with higher levels of SES	8 (36.4)
**Local government interest and commitment to health matters**				
	**Presence of a local health contract (LHC)**			
		No LHC	No LHC	7 (31.8)
		LHC	LHC present	15 (68.2)
	**Presence and internal structure of a local mental health council (LMHC)**			
		Not having an LMHC (No LMHC)	No LMHC created at the time of the intervention	7 (31.8)
		Unstructured LMHC	LMHC classified as unstructured	4 (18.2)
		Structured LMHC	LMHC classified as structured	11 (50)
**Experience in mental health project/policy (EXPInMH)**				
	No (No EXPInMH)		No experience	7 (31.8)
	Yes (EXPInMH)		Experience in conducting projects in mental health	15 (68.2)

**Table 2:** Characteristics of the promotion and its implementation (promotion-related characteristics).

Variables and modalities (names in MCA if different)				Description		Frequency, n (%)
**Delegates degree of understanding of and experience with mental health project as health professionals and coordinators of local mental health council (LMHC)**						
	**Delegate was a health professional**					
		No (No health prof)	Delegate was not a health professional		10 (45.5)	
		Yes (health prof)	Delegate was a health professional		12 (54.5)	
	**Delegate was the coordinator of the LMHC**					
		No (no coordinator LMHC)	Delegate was not the coordinator of an LMHC		11 (50)	
		Yes (coordinator LMHC)	Delegate was the coordinator of the LMHC		11 (50)	
**Provision of additional resources**						
	No (no added resources)		No additional resources were provided		11 (50)	
	Yes (added resources)		Promotion at the local level was provided with extra financial or human resources		11 (50)	
**Set-up of an ad-hoc working group**						
	No (no working group)		Delegate worked mainly alone		13 (59.1)	
	Yes (working group)		Ad-hoc group created locally to help with the promotion implementation		9 (40.9)	
**Involvement of general practitioners (GP) (promotion arm)**						
	Promotion arm including GP (Promo GP)		“Enhanced promotion group” with promotion by the locality and through GP waiting rooms		13 (59.1)	
	Promotion by the locality only (Promo locality)		“Simple promotion group” with promotion by the locality only		9 (40.9)	
**Intensity and type of promotion**						
	Small & traditional promotion		The number of promotional actions was below 5 and only traditional means were used		6 (27.3)	
	Large & traditional promotion		The number of promotional actions was above 5 and only traditional means were used		11 (50)	
	Large & digital promotion		The number of promotional actions was above 5 and digital means (websites, social media) were used		5 (22.7)	

**Table 3:** The outcome variable of the promotion: Utilization rate of StopBlues.

Utilization rate of StopBlues (utilization rate) and its modalities	Description	Frequency, n (%)
Low	Rate < 25 per 100,000 residents or number of active users < 10 (for rate above 25 per 100,000 residents)	6 (27.3)
Medium	Rate ≥ 25 and < 50 per 100,000 residents or number of active users < 20 (for rate above 50 per 100,000 residents).	9 (40.9)
High	Rate ≥ 50 per 100,000 residents and number of active users ≥ 45.	7 (31.8)

### Characteristics of the Pre-existing Context

#### The Level of Urbanization and Socioeconomic Context Of The Area

The geographic area and SES of the localities could have an impact on the utilization rate of SB. As a result, two characteristics were considered: the French Deprivation Index (FDep) and the urban unit, defined by the National Institute for Statistics and Economic Studies as a continuously built-up area with a minimum population of 2000 residents [41-45]. Localities were assigned a low or high SES, based on their FDep quintile: localities in quintiles 1 and 2 (least disadvantaged) were considered high SES, and those in quintiles 3 to 5 were considered low SES. Based on the combination of those two variables, the localities were grouped into three modalities: a rural area with low SES, an urban area with low SES, and an urban area with high SES (only one rural locality, in which the principal place of promotion was an urban unit, was characterized by high SES).

Considering the small number of localities, the number of categorical variables had to be limited and kept as low as possible. The goal was to ensure that the MCA did not lead to misinterpretation with the presence of rare variable modalities being disproportionately weighted in the model [37,46].

The below characteristics were collected through a questionnaire that was distributed to the delegates during the first pre-experimentation meeting and sent out by email to those who were absent (Multimedia Appendix 3).

#### Local Government Interest and Commitment to Health Matters

##### The Presence of a Local Health Contract

Local health contracts (LHCs) were created in 2009 as a roadmap with the objective of reducing health inequalities within a given territory by associating all the relevant local health actors around local governments and the regional health agency (RHA). The latter is the public administrative body of the French State responsible for implementing health policies at the regional level [47,48]. As a result, the presence of an LHC in a locality was considered a good indicator of local government interest and commitment to health matters.

##### The Presence and Internal Structure of a Local Mental Health Council

Local mental health councils (LMHCs), similarly to LHCs, bring together local health and social actors in order to tackle inequalities in the field of mental health by developing and implementing public policies [49]. One of the main goals of LMHCs is to improve mental health through community actions. Therefore, we hypothesized that their presence would have a positive impact on the implementation of the promotion [50].

However, there are no formal requirements on how these structures should run their activities. In fact, where present, there was great variability in the level of their respective internal structure. Thus, a distinction had to be made between “structured” LMHCs, ones that had a roadmap, translated into concrete actions and annual targets displayed in annual reports, and “unstructured” LMHCs, without specific actions and targets. Again, in order to keep the number of analyzed variables low, the presence and internal structure of LMHCs were merged into a single variable. Localities were categorized as not having an LMHC, having an unstructured LMHC, and having a structured LMHC.

#### The Experience in Mental Health Project/Policy

We also considered whether or not the locality had previous experience with conducting projects in mental health, particularly within the past six months [51]. Indeed, the existing literature indicates that the implementation of CBHPs requires a wide range of skills, notably in communication and management. These core competencies and capacities can be built and strengthened through multiple experiences [20].

#### The Delegates Degree of Understanding of and Experience With Mental Health Projects, as Health Professionals and Coordinators of LMHC

Two characteristics concerning the delegate profiles were also considered in the analysis: whether they were health professionals and, where possible, whether they were coordinators of the LMHC. The hypothesis behind this was that the delegates who received their initial education in health and those who were directly involved in the design and implementation of mental health programs would have more experience in conducting mental health projects. We also assumed that they would benefit from a larger network in the field [22,52].

### Characteristics and Implementation of the Promotion

The next four characteristics were based on data collected through a web-based questionnaire that was sent to all the delegates (Figure 1; Multimedia Appendix 4).

#### The Provision of Additional Resources

We presumed that the availability of resources is an important factor that can influence the outcome of an intervention [21,22]. This characteristic therefore comprised all additional resources—human or financial—provided by local governments or RHAs for the implementation of the promotion.

#### The Set-Up of an Ad-Hoc Working Group

No compulsory guidelines for the implementation were given; however, interestingly, some localities put in place dedicated working groups. We hypothesized that the creation of a working group was indicative of long-term assimilation of best practices on collaborative processes, including the ability to co-construct projects with extended networks of partnerships [21,53]. We were interested to see if the presence of working groups could be associated with higher utilization rates.

#### The Involvement of GPs

Because previous studies have shown that the involvement of GPs could positively influence the outcome of an intervention with a focus on primary prevention [16,17,54], the participation of GPs in the promotion was introduced in the analysis. This was dependent on the promotion arm of the trial to which the localities were assigned.

#### The Intensity and Type of Promotion

We hypothesized that the effectiveness of a promotion would depend on the number of promotional actions developed: the more actions put in place, the more effective the promotion would be. However, effectiveness could also depend on the variety of these actions. As a result, we postulated that the use of digital channels (localities website, social media, etc) for promotion would have a positive impact on effectiveness because it targeted the users of eHealth tools directly.

The modalities were built according to the number and type of promotional actions put in place. Therefore, if there were fewer than five actions, the promotion was classified as “small,” and if there were five or more actions, the promotion was classified as “large.” Regarding the type of actions put in place, when conventional and paper-based promotion materials, such as flyers, posters, and leaflets were used, the promotion was classified as “traditional.” Meanwhile, when three and more digital means (websites, newsletters, social media, etc.) were used to promote SB, the promotion was classified as “digital.”

Because no localities with fewer than five promotional actions (small) used three or more digital means, the localities were divided into three categories as follows: small and traditional, large and traditional, and large and digital.

#### The Outcome of the Promotion: Utilization Rate of Stopblues

We assessed the effectiveness of the promotion of SB through SB usage frequency using GA during the two-year experimentation period. Indeed, previous studies have shown that information from GA can be used to evaluate promotional campaigns, determine the geographic distributions of users, and analyze their use of online tools [55-57]. Data were extracted at month 24 directly from GA. This provided general data regarding the users: number (active and new) and location (country and city).

For the purpose of the analysis, the utilization rate variable was categorized as follows: low (< 25 per 100,000 residents or number of active users < 10), medium (between ≥ 25 and < 50 per 100,000 residents or number of active users < 20), and high (≥ 50 per 100,000 residents and number of active users ≥ 45).

### Statistical Analyses

The MCA was performed using all the characteristics of the pre-existing context and those that derived from the program itself (the promotion and its implementation) as active variables. The latter contributed to the construction of a multiple-dimensional coordinate system. Only the first two dimensions were considered. All the variables and individuals were then displayed as plot points in the resulting two-dimensional coordinate system. The outcome variable, the utilization rate, was added to the MCA as a supplementary variable to distinguish whether groups of localities and their implementation characteristics were associated with different levels of utilization rate. Finally, the strength of the correlation between the variables was obtained by running a Pearson correlation test, using the x- and y-dimensional axis scores of the variable modalities produced by the MCA (the categorical variables were transformed into continuous ones). Only correlations above 0.50 (|*r*|≥0.50) (moderate positive or negative correlation) were considered meaningful for the purpose of the analysis [58], and the statistical significance value was set at *P*<.05. All statistical analyses were performed using packages: *FactoMineR* (version 2.3), *Factoshiny* (version 2.3), *Factoextra* (version 1.0.6), and *Ade4* (version 1.7-16) [59-62] from the software R version 3.6.3 (R Foundation).

### Ethical Considerations

The study was granted ethical approval by the relevant ethics committees: the French National Institute for Health and Medical Research (INSERM; approval 15-240 on July 7, 2015), the French Advisory Committee for Data Processing in Health Research (approval 15-793 on September 30, 2015), and the French Data Protection Authority (decision DR-2016-421 on November 3, 2016) [29].

At the user level, the research, its purpose, and outcomes were described in an introductory section, and users had to acknowledge their participation in the intervention by signing a written informed consent. They were also informed that they could withdraw their consent at any time. At the locality level, all local governments signed a convention with the INSERM promoter.

## Results

### Multiple Correspondence Analysis: General Features

The analysis was performed on 22 localities out of the 27 included in the two arms of the CRCT promoting SB: 9/13 (69.2%) and 13/14 (92.9%) in the simple and the enhanced promotion groups, respectively. Three localities dropped out, and two failed to provide data regarding the characteristics necessary for the analysis. Among those localities that dropped out and did not complete the study, two abandoned the study before the beginning of the trial, citing political difficulties and excessive workload, while the third locality terminated its participation when the appointed delegate left her position and was not replaced.

**Figure 2:**
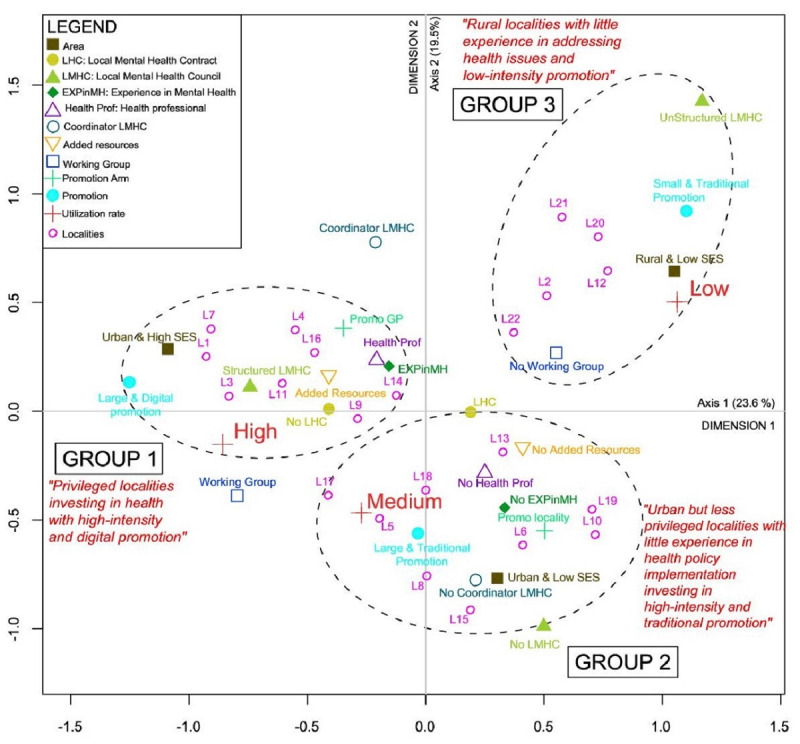
Biplot of the explored variable modalities and typology of the localities.

The total inertia of the MCA model was equal to 1.3. The first two dimensions accounted for 43.1% of the cumulative projected inertia: 23.6% of projected inertia (inertia = 0.307/1.3) for the first dimension (dimension 1) and 19.5% (inertia = 0.254/1.3) for the second (dimension 2) and displayed distinct groups (Figure 2). These dimensions were therefore considered the most relevant for the analysis.

The contributions of the variables for the first two dimensions are presented in Table 4. The closer the value is to 1, the more the variable contributes to the definition of the dimension (1 being the maximum value). The values above the inertia are considered high and meaningful.

**Table 4:** Contribution of active variables.

	Dimension 1 (axis 1/x-axis)		Dimension 2 (axis 2/y-axis)		Mean
Name of the variables	Discrimination	Contribution (%)	Discrimination	Contribution (%)	
Level of urbanization and socioeconomic context of the area	*0.766^a^*	24.976	*0.357^a^*	14.061	0.562
Presence of a local health contract (LHC)	0.078	2.543	0.000	0.000	0.039
Presence and internal structure of a local mental health council (LMHC)	*0.603^a^*	19.661	*0.686^a^*	27.019	0.645
Experience in mental health project/policy	0.052	1.695	0.092	3.623	0.072
Delegate was a health professional	0.052	1.695	0.067	2.639	0.060
Delegate was the coordinator of the local mental health council	0.045	1.467	*0.604^a^*	23.789	0.325
Provision of additional resources	0.168	5.478	0.027	1.063	0.098
Set-up of an ad-hoc working group	*0.440^a^*	14.346	0.104	4.096	0.272
Involvement of GPs	0.175	5.706	0.210	8.271	0.193
Type and intensity of promotion	*0.688^a^*	22.432	*0.392^a^*	15.439	0.540
Total	3.067	100.000	2.539	100.000	2.803
% of projected inertia	23.580		19.530		21.550
Inertia	0.307		0.254		

^a^The italicized values are considered high and meaningful (above the inertia).

### Three Different Profiles of Localities and Their Respective Promotion Implementation

The MCA identifies three distinct groups of localities characterized by different implementation profiles (Figure 2).

In the MCA plot, the statistical strength of the relationship between the variable modalities is determined by their spatial proximity and their location within the quadrants. The closer the variables are to each other, the stronger the relationship among the localities sharing these features. At the same time, the greater the distance of a modality from the intersection of the axes, the stronger its significance in the interpretation of results.

Four variables specifically helped shape the groups (Tables 4 and 5): the level of urbanization and socioeconomic context of the area in which the localities were situated; the presence and internal structure of LMHCs; the intensity and type of promotion implemented; and the set-up of an ad hoc working group.

**Table 5:** Correlations between meaningful variables in both dimensions.

Variables	Level of urbanization and socioeconomic context of the area	Presence and internal structure of a local mental health council	Delegate was the coordinator of the local mental health council	Set-up of an ad-hoc working group	Type and intensity of the promotion implemented in the localities	SB utilization rate
Level of urbanization and socioeconomic context of the area	1					
Presence and internal structure of a local mental health council	Dimension 1: *r*=0.626 *P*=.002; dimension 2: *r*=0.291 *P*=.02					
Delegate was the coordinator of the local mental health council	Dimension 1: *r*=0.289 *P*=.22; dimension 2: *r*=0.073 *P*=.14	Dimension 1: *r*=0.061 *P*=.82; dimension 2: *r*=0.711 *P*<.001				
Set-up of an ad-hoc working group	Dimension 1: *r*=0.448 *P*=.29; dimension 2: *r*=0.252 *P*=.26	Dimension 1: *r*=0.499 *P*=.02; dimension 2: – *r*=0.027 *P*=.76	Dimension 1: – *r*=0.092 *P*=.68; dimension 2: *r*=0.092 *P*=.68			
Type and intensity of the promotion implemented in the localities	Dimension 1: *r*=0.651 *P*=.001;dimension 2: *r*=0.216 *P*=0.33	Dimension 1: *r*=0.674 *P*<.001; dimension 2: *r*=0.659 *P*=.002	Dimension 1: – *r*=0.057 *P*=.83; dimension 2: *r*=0.265 *P*=.22	Dimension 1: *r*=0.712 *P*<.001; dimension 2: *r*=0.235 *P*=.60		
SB utilization rate	Dimension 1: *r*=0.790 *P*<.001; dimension 2: *r*=0.436 *P*=.04	Dimension 1: *r*=0.650 *P*<.001; dimension 2: *r*=0.321 *P*=.27	Dimension 1: *r*=0.000 *P*=1.0; dimension 2: *r*=0.000 *P*=1.0	Dimension 1: *r*=0.660 *P*<.001; dimension 2: *r*=0.534 *P*=.01	Dimension 1: *r*=0.651 *P*<.001; dimension 2: *r*=0.676 *P*=.003	1

### Privileged Localities Investing in Health With High-Intensity and Digital Promotion

A first group (Group 1 in Figure 2) located in the upper left quadrant is comprised of the eight most urbanized and wealthiest localities (L1, L3, L4, L7, L9, L11, L14, and L16).

These localities were characterized by strong supportive environments with solid networks of local health service organizations, such as LMHCs, and significant experience in mental health project management and policy implementation. Moreover, the delegates were mostly health professionals and coordinated the LMHCs.

The promotion pattern of SB in those localities was characterized by the investment of additional resources—financial, material, or human—and a working group aimed at coordinating the local implementation. They also included digital strategies in the promotion. Besides, they tended to more frequently involve GP waiting rooms as promotion channels for SB.

### Urban but Less Privileged Localities With Little Experience in Health Policy Implementation Investing in High-Intensity and Traditional Promotion

The second group (see Group 2 in Figure 2), located in the lower quadrants of the plot, includes ten localities (L5, L6, L8, L10, L13, L15, L16, L17, L18, and L19).

These were mostly situated in an urban area with low SES and had no experience in conducting projects in mental health, indicating that the environment was less supportive. This was illustrated notably by the absence of an LMHC. Additionally, in most cases, the appointed delegates from these localities were not health professionals. Yet, they still managed to put in place a large-scale traditional promotion, backed by the set-up of a working group.

### Rural Localities With Little Experience in Addressing Health Issues and Low-Intensity Promotion

This third group (see Group 3 in Figure 2), located on the upper right quadrant, is comprised of five localities (L2, L12, L20, L21, and L22) mostly situated in rural areas with low SES levels. Here, the environment was the least supportive, as illustrated by the fact that their LMHC was not structured with a clear roadmap. The localities also lacked experience in conducting mental health projects, and their appointed delegates were not health professionals.

In this group, the promotion pattern of SB was characterized by no investment of additional resources. They implemented a low-intensity promotion, defined by a few actions put in place, and did not create a working group for the purpose of the promotion.

### Association Between Localities Groups and Utilization Rate of SB

The addition of the utilization rate to the MCA as a supplementary variable displayed a strong relationship between the profiles of the localities and the utilization of SB (Figure 2).

The privileged localities investing in health with high-intensity digital promotion were associated with the highest utilization rates. The urban, but less privileged localities, with little experience in health policy implementation and investing in high-intensity traditional promotion, were associated with medium utilization rates. Finally, rural localities with little experience in addressing health issues and low-intensity promotion were associated with the lowest utilization rates.

Besides, correlations between the meaningful variables, displayed in Table 5, were determined from the coordinate x- and y- dimensional axis scores. In dimension 1, the SB utilization rate was strongly correlated with the level of urbanization and socioeconomic context of the area, the LMHC (presence and internal structure), the set-up of an ad-hoc working group, and the type and intensity of the promotion implemented in the localities. In dimension 2, the SB utilization rate was also strongly correlated with the type and intensity of the promotion and, to a lesser extent, with the set-up of an ad-hoc working group.

## Discussion

### Principal Findings

Three distinct promotion patterns emerged, according to the profiles of localities, that were associated with the utilization rates of SB. These patterns were identified in the MCA, where, given the small number of localities included in the analysis, only correlations above 0.50 (|*r*|≥0.50) were considered significant. From highest to lowest utilization rates, the promotion patterns are listed as follows:

The urban “privileged localities investing in health” that implemented high-intensity promotion with a digital component demonstrated a greater capacity to take ownership of the project, understand its ins and outs, and anticipate potential challenges.

The “urban but less privileged localities” that, despite having relatively little experience in health policy implementation, managed to implement a high-intensity, traditional promotion.

The “rural localities with little experience in addressing health issues” that implemented low-intensity promotion could not overcome the challenges associated with their context-based characteristics (low SES, rural setting, and unstructured LMHCs, in particular).

Overall, contextual characteristics related to local government interest and commitment to health matters and the experience in mental health policy implementation were decisive to the success of the intervention and utilization of SB. Localities that were deeply involved in the structuring of health programs and with greater experience managed to implement promotional campaigns that resulted in higher utilization rates. The presence of dedicated local health service organizations to help with the implementation of public health interventions was crucial, although not sufficient to guarantee their success. Indeed, they also needed to be structured, with a roadmap that was translated into concrete actions and annual targets. As previously noted, this shows that the collaboration between local partners represents a fundamental parameter to tackle public health issues within a community [63]. Hence, dedicated structures, like LHCs and LMHCs, could not handle problems when pre-existing shortcomings, such as lack of resources or local partners, were not addressed in the first place [48].

Localities where working groups were implemented had higher SB utilization rates, indicating the importance of being able to not only mobilize existing partners but also organize the implementation processes of an intervention. Stronger networks and a history of partnerships between local stakeholders could more naturally lead to a greater willingness to work together and, thus, collaborate in working groups [21,53,63].

Finally, the involvement of GPs in the promotion was globally associated with higher SB utilization rates. More precisely, 75% of the localities characterized by high utilization rates were included in the enhanced promotion group that involved GPs. This is consistent with previous literature findings regarding the important role of primary care in the prevention of suicide [16,17,54].

Among the findings related to the program itself, the type of promotion was particularly noteworthy. The implementation of a digital promotion was associated with the highest SB utilization rates. Other researchers have already pointed out the utility of digital marketing and, more specifically, the use of social media to promote health [23,64,65]. In effect, it seems logical to use the internet to promote eHealth tools. This is notably true when considering eHT in mental health care. Indeed, because of the stigma associated with mental illness, people with poor mental health are more likely to use the internet to find information and possible solutions to their problems [66-68]. Likewise, social media have many advantages such as cost-effectiveness, 24/7 availability, and potential broad audience reach. They also allow for interactive engagement [69,70].

In addition, those who support the utilization of eHT in mental health have previously acknowledged the benefits of employing digital channels for user engagement [71,72]. However, notwithstanding the growing body of literature on social media, the evaluation of the effectiveness of their utilization in health is lacking [73,74]. In fact, to our knowledge, this is the first study in a French setting that confirms the potential of social media for the promotion of eHT.

Another important factor to be considered is the population targeted by the promotion. Regarding the sociodemographic context of the implementation, we notably found that the more deprived and rural the localities were, the lower the utilization rates of SB. Previous studies have highlighted similar results, where promotion interventions were less effective among populations with lower SES [27,28,75]. Two reasons for this can be put forward: First, these populations generally lack access to health information and do not have the ability to interpret and process the information [27]. This explains why the segments of a population with the lowest incomes tend to be less likely to benefit from preventive care. Second, they are less likely to use eHealth tools, such as mobile health apps, either because of lack of adequate access or lack of skills and training [1,76,77].

Several studies have stressed that the effectiveness of health promotion interventions varied between rural and urban settings [78-80]. Indeed, rural communities face many specific challenges related to the difficulty of accessing a range of health care services and an increasing shortage of health professionals [71,78]. However, to have a deeper understanding of the differences between rural and urban areas in terms of SB utilization rates, three factors should be carefully examined. Firstly, rural localities tend to cover larger and more remote areas; therefore, the implementation of a homogenous and uniform promotion is more challenging [71,79,81]. Secondly, while digital health promotion could be a solution to engage and reach the rural populations [71], these areas are still characterized by poorer broadband access, which can limit the adoption of eHT [82,83]. Thirdly, rural populations tend to be older than urban ones [84,85]. Previous studies have indicated that older adults have more limited access to technology in general and, consequently, tend to possess poorer digital health literacy skills than younger age groups [86-88].

Thus, lower digital literacy levels are amplified through the joint effect of SES and age in rural localities. This supports the idea raised by Frohlich and Potvin that population-level approaches to eHT should be complemented with interventions focusing directly on vulnerable populations in order to alleviate the digital divide [89].

### Limitations

Limitations in this study are primarily due to the sample size of localities, the data used, and the method of the MCAs.

The analysis was conducted on a sample size of only 22 localities. While no rule exists regarding the minimum number of observations needed to run an MCA, small sample sizes affect the reliability and interpretation of the results [38,46]. However, Di Franco [46] indicates that a sufficient sample consists of 20 observations per single active categorical variable, and CRCTs that involve less than 30 clusters [90,91] are also commonly used in the assessment of interventions in prevention.

Other limitations of this study are related to the data used to perform the MCA. The first is associated with how the delegates responded to the self-report questionnaires that were used to collect data on the type and intensity of promotion that they implemented in their locality. Possible classical information biases can arise, such as social desirability or recall biases. Social desirability bias is the tendency to overreport more socially desirable attributes and behaviors and underreport attitudes that are perceived as socially undesirable [92]. In recall bias, the study participants do not remember their previous actions related to the question that was asked [93].

A second limitation involves the use of GA to obtain the user statistics. Because GA models are calculated at the country level, Google states that metrics are not always accurate, “particularly for campaigns that target small geographical areas, such as a single city or zip code” [94]. However, one can assume that this lack of accuracy is evenly distributed across the arms of the trial.

Regarding the MCA method itself, only correlations |*r*|≥0.50 were considered significant, whereas the significance threshold for the coefficient correlation in exploratory and MCAs can be lowered to |*r*|≥0.30 and still be meaningful [95,96]. However, considering the small number of localities included in our analysis, we decided to keep the threshold at |*r*|≥0.50 in order to avoid any misinterpretation [97].

On a more general level, the benefits of the MCA utilization to transform qualitative data into quantitative data can also be its pitfall. Indeed, by simplifying the contained data, we can also lose the complexity of information and not fully understand the ins and outs of the situation [38,98]. This is why further analysis based on a more qualitative approach should be carried out in order to complement the methodology and deepen the findings.

### Conclusions

The use and dissemination of the StopBlues eHealth tool depended heavily on the promotion that was conducted.

The urban and SES profiles of localities, along with their investment and pre-existing experience in health, appear to be critical for shaping the implementation of the promotion of the SB eHealth tool in terms of intensity and use of digital communication. The more digital channels used, the higher the utilization rates, ultimately leading to the overall success of the intervention.

Digital communication, and more specifically, social media, seem to be powerful tools for engaging hard-to-reach populations. This raises the question of the information and communication technology skills and digital literacy, along with the attitudes towards new technologies, of the professionals in charge of the promotion of eHT. They should be trained in order to be receptive and proactive in the use of eHT to the benefit of the greatest number of people.

Further, to broaden the outreach, other innovative means and promotional strategies should be considered to reinforce and back up promotion campaigns of eHT initiated at the local level. New web-based marketing techniques, where the user could be informed of useful and targeted interventions at the very moment they are “engaging in information-seeking behavior through online search inquiry” [99], could open up entirely new perspectives in the way public health interventions are built. This is particularly the case in the field of mental health, where online information-seeking behaviors are widespread due notably to the fear of stigma [66,67]. However, further research is needed to understand how to avoid eHT becoming a source of social and health inequalities, such that it may be leveraged for social good.

## Appendix

Multimedia Appendix 1 Members of the PRINTEMPS Consortium.

Multimedia Appendix 2 Detailed characteristics of the StopBlues promotion.

Multimedia Appendix 3 The questionnaire was administered to the attendees of the first meeting at M-7 and sent by email to those who were absent. Its goal was to assess the local situation (local health system, presence of health service organization, past experience) and identify the local point person for the promotion.

Multimedia Appendix 4 The web-based questionnaire was designed and sent by email at M10 to all the delegates. The aim was to investigate how the promotion was set up and conducted in the different settings.

## Abbreviations

CBHP: community-based health promotion
CRCT: cluster randomized controlled trial
eHT: eHealth technologies
FDep: French Deprivation Index
GA: Google Analytics
GP: general practitioner
INSERM: French National Institute of Health and Medical Research
LHC: local health contract
LMHC: local mental health council
MCA: multiple correspondence analysis
RHA: regional health agency
SB: StopBlues
SES: socioeconomic status
WHO: World Health Organization
