# Supplementary material for: Conditions for the Successful Integration of an eHealth Tool "StopBlues" Into Community-Based Interventions in France: Results From a Multiple Correspondence Analysis
Source: J Med Internet Res. 2022 Apr 22;24(4):e30218. doi: 10.2196/30218 (PMC9077507; doi:10.2196/30218)
Supplement: Multimedia Appendix 1 [file jmir_v24i4e30218_app1.docx]

## Multimedia Appendix 1: Members of the PRINTEMPS Consortium

The following are members of the PRINTEMPS Consortium: Corinne Alberti, Université de Paris, Unité UMR 1123 ECEVE, INSERM, Paris, France & Hôpital Robert Debré, CIC-EC, Unité INSERM CIC 1426, Assistance Publique-Hôpitaux de Paris, Paris, France; Karine Chevreul, Université de Paris, Unité UMR 1123 ECEVE, INSERM, Paris, France & Health Economics Clinical Research Platform (URC Eco), AP-HP, 1 Place du Parvis Notre-Dame, 75004, Paris, France; Philippe Courtet, Department of Psychiatric Emergency and Acute Care, Lapeyronie Hospital, CHU Montpellier, Montpellier, France & Neuropsychiatry, Epidemiological and Clinical Research, INSERM, University of Montpellier, Montpellier, France; Coralie Gandré, Université de Paris, Unité UMR 1123 ECEVE, INSERM, Paris, France; Bruno Giraudeau, INSERM CIC 1415, CHRU de Tours, Tours, France & Tours University, Nantes University, INSERM SPHERE, U1246, Tours, France; Anaïs Le Jeannic, Unité UMR 1123 ECEVE, INSERM, Paris, France & Health Economics Clinical Research Platform (URC Eco), AP-HP, 1 Place du Parvis Notre-Dame, 75004, Paris, France; Jean-Luc Roelandt, World Health Organization Collaborating Centre for Research and Training in Mental Health, Établissement Public de Santé Mentale Lille Metropole, Lille, Hellemmes, France; Kathleen Turmaine, Université de Paris, Unité UMR 1123 ECEVE, INSERM, Paris, France; Guillaume Vaiva, Department of Adult Psychiatry, CHU Lille, Lille, France & Centre National de Ressources et Résilience pour le Psychotraumatisme (Cn2r Lille Paris), Lille, France; Marie-Amélie Vinet, Unité UMR 1123 ECEVE, INSERM, Paris, France & Health Economics Clinical Research Platform (URC Eco), AP-HP, 1 Place du Parvis Notre-Dame, 75004, Paris, France.
