# Supplementary material for: Conditions for the Successful Integration of an eHealth Tool "StopBlues" Into Community-Based Interventions in France: Results From a Multiple Correspondence Analysis
Source: J Med Internet Res. 2022 Apr 22;24(4):e30218. doi: 10.2196/30218 (PMC9077507; doi:10.2196/30218)
Supplement: Multimedia Appendix 2 [file jmir_v24i4e30218_app2.docx]

Multimedia Appendix 2: Promotion characteristics

| PROMOTION ARMS | | STUDY SAMPLE | | | PROMOTION | | | | | |
| --- | --- | --- | --- | --- | --- | --- | --- | --- | --- | --- |
| **Arm** | **Definition** | **Inclusion (on a voluntary basis)**  *Localities randomly allocated to one of the three arms by minimization* | | | **Who**?  *Party in charge of the implementation*^[[1]](#footnote-1)^ | **What?**  *Health promotion program implemented based on past experience and/or on a list of suggestions provided by the research team* | **How?**  *Materials used during the promotional campaign* | | **Where?**  *Information materials displayed around the perimeter of the locality* | **When?**  *Promotion start and end dates were left to the discretion of each locality* |
|  |  |  |  |  |  |  |  |  |  |  |
|  |  | Baseline localities | Number of dropouts | Number of localities in the analysis |  |  | Materials for implementation² | Materials for promotion² |  |  |
| ARM 1  Control group | 12-month delay in the promotion | 15 | 3 | 0 | The delegate | Range of promotional actions developed | Booklet and technical flyers | Flyers, posters, leaflets, web banner, PowerPoint presentations | In all possible public places, digital spaces and partners' infrastructures identified for the promotion | After April 2019 (M+12) |
| ARM 2 Simple promotion | Promotion by the locality only | 13 | 3 | 9 | The delegate | Range of promotional actions developed | Booklet and technical flyers | Flyers, posters, leaflets, web banner, PowerPoint presentations | In all possible public places, digital spaces and partners' infrastructures identified for the promotion | From October 2017 (M-6) |
| ARM 3 Enhanced promotion | Promotion by the locality and through general practitioner (GP) waiting rooms | 14 | 0 | 13 | - Promotion by the locality: The delegate | Range of promotional actions developed | - Promotion by the locality: booklet and technical flyers | -Promotion by the locality: Flyers, posters, leaflets, web banner, PowerPoint presentations | In all possible public places, digital spaces and partners' infrastructures identified for the promotion | From October 2017 (M-6) |
|  |  |  |  |  | - Promotion through GP waiting rooms:  The research team | Put up posters and leaving flyers and leaflets in waiting areas | - Promotion through GP waiting rooms: presentation letter signed by the main investigator inviting GPs to participate to the study and display promotional materials | -Promotion by the GPs: a basic kit including one poster A3, 60 flyers and 40 leaflets | In practice waiting areas | From March 2018 (M-1) |

1. The delegate was not always working alone on the project, dedicated working groups were established in several localities to help with the implementation of the intervention

   ² Provider of “implementation” materials: Research team (design and printing); Providers of the promotional materials: (1) *Research team* (design and printing for non-customised generic versions); (2) *Locality* (printing for customised versions with the locality logo etc.) [↑](#footnote-ref-1)
