# Supplementary material for: Conditions for the Successful Integration of an eHealth Tool "StopBlues" Into Community-Based Interventions in France: Results From a Multiple Correspondence Analysis
Source: J Med Internet Res. 2022 Apr 22;24(4):e30218. doi: 10.2196/30218 (PMC9077507; doi:10.2196/30218)
Supplement: Multimedia Appendix 3 [file jmir_v24i4e30218_app3.docx]

Multimedia Appendix 3: Questionnaire 1

**Locality, Delegates’ position and background**

- What is your name and what locality are you representing today?
- What is your current position?
- What is your education background?

**Identify the delegates in each locality**

- Are you the designated StopBlues delegate in your locality?
- If not, who is the StopBlues delegate in your locality?

**Organization of the local health systems and existing structures**

- Are your involved in the LMHC (Local mental health council) network?
- If yes, are you the coordinator of the LMHC?
- If not, is the StopBlues delegate from your locality the LMHC coordinator?
- Did your locality contracted an LHC (local health contract)?
- If yes, are you the current coordinator of it?
- If not, does the StopBlues delegate from your locality coordinate the LHC?
- Is your locality part of the Health and Urban Policy Workshop network?
- If yes, do you coordinate it?
- If not, does the StopBlues delegate from your locality coordinate the Health and Urban Policy Workshop network locally?

**Past experience in mental health**

- Over the past year, has your locality implemented some prevention initiatives in the field of mental health?

**Implementation of the StopBlues program**

- Currently, what are the resources on which you or the StopBlues delegate of your locality can rely on to assist you or her/him in the task?
- Do you currently foresee any type of challenges ahead regarding the implementation of the promotion?
- If yes, specify.
- Do you have any comments/questions related to the StopBlues program?
- If yes, specify.

**Involvement and interconnection with local General practitioners (GPs).**

- Do you have a database or a listing of all GPs practicing in your locality?
- Would it be possible for you to contact these GPs and inform them about the StopBlues program?
- If your locality had to choose one group, would it prefer to be included in the enhanced promotion group**?**

## 
