# Supplementary material for: Conditions for the Successful Integration of an eHealth Tool "StopBlues" Into Community-Based Interventions in France: Results From a Multiple Correspondence Analysis
Source: J Med Internet Res. 2022 Apr 22;24(4):e30218. doi: 10.2196/30218 (PMC9077507; doi:10.2196/30218)
Supplement: Multimedia Appendix 4 [file jmir_v24i4e30218_app4.docx]

Multimedia Appendix 4: Questionnaire 2

**Assistance**

- Have you ever received any financial assistance for the implementation of the promotion?
- If yes, from whom and for what purpose precisely?
- Where you assisted by someone during the implementation and conduct of the StopBlues promotion?
- If yes, who was your support and what type of assistance did you receive?

**Start date of the promotion**

- When did you start the promotion in your locality (or when are you planning to start)?
- Did you start the promotion with an official launch event (OR are you planning to start the promotion with an official launch event)?
- If yes, name the event.

**Target audience and end date**

- Did/do you target a specific audience?
- What was/is your target audience and why?
- What is your planned end date (already happened or foreseen)?

**Type of promotion**

- Did/do you have a continuous or intermittent promotion campaign? (OR are you planning to have a continuous or intermittent promotion campaign)
- If you put in place an intermittent promotion campaign, how many cycles did you have (OR how many cycles are you planning to have)?

**Support materials and promotional tools**

- How many materials from the toolkit provided by the research team did/do you use to implement and conduct the promotional campaign in your locality? (List provided)

**Activities put in place**

- Which of these promotional actions did you/do you intend to/ put in place during the promotional campaign in your locality? (List provided)
- Which of these promotional activities have you prioritized (do you intend to prioritize) during your campaign?
